# The alexithymic brain: the neural pathways linking alexithymia to physical disorders

**DOI:** 10.1186/1751-0759-7-1

**Published:** 2013-01-09

**Authors:** Michiko Kano, Shin Fukudo

**Affiliations:** 1 Behavioral Medicine, Tohoku University Graduate School of Medicine, Tohoku University, 2-1 Seiryo-cho, Aoba-ku, Sendai, 980-8575, Japan

**Keywords:** Affect, Alexithymia, Emotional dysregulation, Neuroimaging, Psychosomatic disorders

## Abstract

Alexithymia is a personality trait characterized by difficulties in identifying and describing feelings and is associated with psychiatric and psychosomatic disorders. The mechanisms underlying the link between emotional dysregulation and psychosomatic disorders are unclear. Recent progress in neuroimaging has provided important information regarding emotional experience in alexithymia. We have conducted three brain imaging studies on alexithymia, which we describe herein. This article considers the role of emotion in the development of physical symptoms and discusses a possible pathway that we have identified in our neuroimaging studies linking alexithymia with psychosomatic disorders. In terms of socio-affective processing, alexithymics demonstrate lower reactivity in brain regions associated with emotion. Many studies have reported reduced activation in limbic areas (e.g., cingulate cortex, anterior insula, amygdala) and the prefrontal cortex when alexithymics attempt to feel other people’s feelings or retrieve their own emotional episodes, compared to nonalexithymics. With respect to primitive emotional reactions such as the response to pain, alexithymics show amplified activity in areas considered to be involved in physical sensation. In addition to greater hormonal arousal responses in alexithymics during visceral pain, increased activity has been reported in the insula, anterior cingulate cortex, and midbrain. Moreover, in complex social situations, alexithymics may not be able to use feelings to guide their behavior appropriately. The Iowa gambling task (IGT) was developed to assess decision-making processes based on emotion-guided evaluation. When alexithymics perform the IGT, they fail to learn an advantageous decision-making strategy and show reduced activity in the medial prefrontal cortex, a key area for successful performance of the IGT, and increased activity in the caudate, a region associated with impulsive choice. The neural machinery in alexithymia is therefore activated more on the physiologic, motor-expressive level and less in the cognitive-experiential domains of the emotional response system. Affects may play an important role in alleviating intrinsic physiologic reactions and adapting to the environment. Deficient development of emotional neural structures may lead to hypersensitivity to bodily sensations and unhealthy behaviors, a possible mechanism linking alexithymia to psychosomatic disorders.

## Introduction

The alexithymia concept was introduced by Sifneos [1] from the clinical observation of patients with classic psychosomatic diseases, in which they failed to respond to dynamic psychotherapy. It was conceptualized as encompassing a cluster of cognitive traits including difficulty identifying feelings, difficulty describing feelings to others, externally oriented thinking, and a limited imaginative capacity [2]. Research investigating the alexithymia construct has advanced rapidly, owing to the development of the self-reported, 20-item, Toronto Alexithymia Scale (TAS-20) [3,4]. The TAS-20 provides investigators with a reliable, validated, and common metric for measuring the construct [5,6]. Individuals with a TAS-20 total score of ≥61 are considered alexithymic, and those with a score of <51 are considered nonalexithymic [7]. The TAS-20 comprises three subscales: difficulty identifying feelings, difficulty describing one’s feelings, and presence of externally oriented thinking [5,6]. Alexithymia was assessed by the TAS-20 in the studies addressed in this article. However, we note some researchers question whether self-report measures of alexithymia, including the TAS-20, provide a valid assessment of the construct and recommend the use of multiple methods of measurement [8,9]. Various instruments have been developed, such as observer-rated measures including the modified Beth Israel Hospital Psychosomatic Questionnaire (BIQ), the Observer Alexithymia Scale, a set of Rorschach variables and the Toronto Structured interview for Alexithymia [10]. The Levels of Emotional Awareness Scale (LEAS) is also a self-report instrument to elicit written emotional response and often compared to the TAS-20 [11].

Although alexithymia is personality trait independent from clinical diseases, a high rate of alexithymia has been reported in several medical conditions and psychiatric disorders such as asthma, hypertension, and chronic pain [5,6]. In particular, it is an important phenomenon in a relevant subgroup of patients with functional somatic disorders including functional gastrointestinal disorders [12-15]. It is also associated with increased mortality [16,17]. The prevalence of alexithymia is approximately 10% in the general population, whereas a high rate of alexithymia (approximately 40–60%) is reported among patients with psychosomatic disorders [7,12]. Alexithymia is considered a key element associated with the psychosomatic process [18,19] and appears to play a role in neurologic causes of physical illness [20].

The mechanism underlying alexithymia and its association with physical illness remain to be elucidated. Nevertheless, several pathways have been suggested, as follows: alexithymia leads to organic disease via physiologic or behavioral mechanisms; alexithymia leads to illness behavior (physical symptoms, disability, and excessive health care use) via cognitive or social mechanisms; physical illness leads to alexithymia; both alexithymia and physical illness result from sociocultural or biologic factors [20].

Physiologically, because the main problem of alexithymic personality traits is considered to be deficit in affect regulation, it is hypothesized that alexithymia constitutes a longstanding risk factor for imbalances in the autonomic nervous system and the neuroendocrine system [15]. The few immunology studies that have been conducted have found that alexithymia is associated with poor immune status [5], and a number of studies have found that alexithymia is related to increased levels of resting (tonic) sympathetic or cardiovascular activity [5]. Recent progress in neuroimaging studies on alexithymia has provided important information on the neural basis of alexithymia. In particular, a number of studies have examined brain responses underlying affect dysregulation in alexithymia [21-36]. In addition to dysfunction of the autonomic nervous system and neuroendocrine system, we now consider that the alexithymic brain may cause a variety of psychosomatic disorders. Here we review recent neuroimaging findings related to emotional experience in alexithymia, including those from three of our own studies on emotional processing, visceral processing, and emotion-guided decision-making, and discuss a possible neural mechanism linking alexithymia and psychosomatic processes. In contrast to the many studies focused on the personality dimensions of alexithymia, we would like to provide a unique review of alexithymia from the view point of the linking alexithymia to somatic conditions.

### Socio-affective processing in alexithymia

Research has repeatedly shown impairment of emotional processing in alexithymia, that is, in the recognition of basic emotions associated with emotionally valenced sentences, words, faces, and scenes [37-39]. Therefore, in our first experiment we conducted a brain imaging study of facial expression processing in alexithymia, in which regional cerebral blood flow (rCBF) was measured by positron emission tomography (PET) [22]. Subjects performed gender discrimination of angry, sad, and happy facial expressions, as well as neutral faces, during PET scanning; thus, the emotional component of stimuli was assessed implicitly. Alexithymics exhibited lower rCBF in the right hemisphere in the inferior and middle frontal cortex, orbitofrontal cortex, superior temporal gyrus, inferior parietal cortex, and fusiform gyrus compared to nonalexithymics. In addition, alexithymics showed higher rCBF in the left hemisphere in the superior frontal cortex, inferior parietal cortex, superior temporal gyrus, and cerebellum compared to nonalexithymics. There was no significant difference in rCBF between alexithymics and nonalexithymics when they looked at neutral faces. Moreover, the dorsal anterior cingulate cortex (dACC) and bilateral insula were less activated in alexithymics in response to angry faces compared to their responses to neutral faces. The process underlying the recognition of emotion in faces has been characterized [40]. Lateral portions of the inferior occipital gyrus, fusiform gyrus, and superior temporal gyrus are disproportionately activated in facial processing [40]. The amygdala is particularly activated in response to facial expressions of fear, and the orbitofrontal cortex and ACC are activated by facial expressions of anger [40]. The right primary and secondary somatosensory areas, insula, and anterior supramarginal gyrus are involved in the recognition of emotion [40].

Therefore, our above findings indicate that alexithymics show dampened responses in emotional facial processing regions in the right hemisphere and increased responses in those areas in the left hemisphere. In addition, the dACC has been suggested to play a crucial role in conscious awareness of emotion [41,42], and dysfunction in the dACC has been proposed to be one of the main mechanisms of alexithymia [8]. The anterior insula appears to be critically involved in subjective emotional experience and awareness of the internal bodily state [43-46]. Hence, in particular, alexithymia shows less activation in brain areas associated with emotional awareness during the viewing of facial expressions.

With regard to socio-affective processing, a number of studies have demonstrated different brain responses in areas associated with emotional processing. For example, activity in the amygdala is reduced in alexithymics during the viewing of a masked, sad face [31,34], in viewing pictures evoking anxiety and disgust [47], in response to threatening emotional actions [30], and with respect to positive autobiographic recall [29]. In addition, it has been reported that a female cocaine user showed a negative correlation between activity in the right amygdala and alexithymia score in script-guided imagery of neutral or stressful situations [48]. Reports of individuals with high-functioning autism/Asperger syndrome also demonstrate that alexithymia score is negatively correlated with activity in the left amygdala during the rating of affective pictures [49]. The amygdala plays a central role in several aspects of emotional processing, such as the recognition of both positively and negatively valenced emotional stimuli [50,51]. Less activation in the insula is also reportedly associated with alexithymia in response to a masked, sad face [31] and in response to affective pictures among individuals with autism [49]. Many studies have reported alteration in activity in the ACC during socio-affective tasks. Among these, 50% report a negative correlation, 40% report a positive correlation, and 10% show both types of associations (ie, high-alexithymia individuals showed more activation of the rostral ACC, whereas low-alexithymia individuals showed more activation of the dACC) [30]. Activity in the dACC has been reported to be increased in response to implicit presentation of facial expressions [52], in contrast to our results. These discrepancies may be related to experimental variables including tasks and stimuli. For example, higher activation in the dACC and lower activation in the amygdala has been shown to be associated with alexithymia in the same experiment. Depending on the context of the socio-affective task, dACC activity may increase or decrease. The function of the dACC may not always be associated with emotional awareness; sometimes the ACC interacts with other parts of the brain to sustain self-regulation of one’s own emotional state. The above, taken together with our results, provide accumulating evidence for dysfunction in regions related to socio-affective processing in alexithymia.

### Interoceptive arousal in alexithymia

#### Somatosensory amplification

Another well-reported characteristic of alexithymia is somatosensory amplification. Research has shown that patients with chronic pain have higher levels of alexithymia than control subjects, and alexithymia is associated with overreporting of physical symptoms [13,20,53-55]. An association between alexithymia and increased pain intensity and sensitivity to experimentally induced pain has been demonstrated [56-58]. The degree of alexithymia correlates with the score of somatosensory amplification [59]. Therefore, alexithymics might perceive signals from the body in an aberrant manner such that low-intensity stimulation is perceived as high intensity. In particular, functional gastrointestinal disorders, such as irritable bowel syndrome, characterized by chronic gastrointestinal symptoms with no biochemical abnormalities, are associated with alexithymia [14,60,61].

Therefore, in our second study, we investigated brain responses to visceral stimulation induced by colonic distension between alexithymics and nonalexithymics. During colonic distension, greater activation was observed in the pregenual ACC, right insula, and midbrain in subjects with alexithymia [19]. The TAS-20 score correlated positively with both activity in the right insula and orbital gyrus and adrenaline levels in the blood in response to stimulation. Subjects with high scores for difficulty identifying feelings perceived strong pain, urgency for defecation, stress, and anxiety.

Visceral stimulation, especially by colonic distension, has been reported to elicit activation of various brain areas including the posterior insula, prefrontal cortex (PFC), ACC, and brainstem periaqueductal gray (PAG) [62-64]. A detailed, dynamic representation of particular internal bodily states and the mapping of each interoceptive state occurs within the orbitofrontal and right insular cortices [65,66]. In the cingulate cortex, the pregenual ACC responds to visceral stimulation and is associated with the perception of secondary pain, which is characterized by greater unpleasantness [67]. Electrical stimulation of the insula has been reported to elicit changes in blood pressure, heart rate, respiration, gastric motility, peristaltic activity, salivation, and adrenaline secretion [68]. In addition, the orbitofrontal cortex has been shown to receive robust sensory input and to act as an internal environmental integrator that coordinates behavioral, autonomic, and endocrine responses [69]. The brainstem is known to control ascending nociceptive input and nuclei such as the rostral ventromedial medulla and PAG, which are able to both inhibit and facilitate nociceptive responses. In particular, activation of the right PAG has been reported to correlate with anxiety during visceral stimulation but not somatic stimulation [70]. Therefore, it may be assumed that activation of brain areas associated with alexithymia represents afferent representation of bodily states and efferent autonomic and endocrine responses that accompany it. Alexithymics may be more aroused by interoception of unpleasant feelings than nonalexithymics, thereby displaying more autonomic responses.

The evidence supports the somatosensory amplification hypothesis of alexithymia. There have been no other imaging studies investigating pain or interoception processing in alexithymia. However, a few, in which interoceptive or physiologic arousal was induced, have provided suggestive findings. Moriguchi et al. examined brain responses of alexithymics during visual perception of pictures depicting human hands and feet in painful situations [26]. In this task assessing empathy to others’ pain, alexithymics showed greater brain activity in the posterior and anterior insula. The posterior insula is the primary endpoint of the interoceptive pathway, which represents sensory aspects from the body including pain, touch, and visceral sensation [43,65]. The information in the posterior insula is rerepresented in the right anterior insular cortex, which provides subjective awareness of the feelings from the body, that is, metarepresentation of interoception [43,65]. Other brain areas in the pain matrix, such as the dorsolateral PFC, dorsal pons, and left caudal ACC, which are associated with modulation of pain sensations, are less activated, and the pain rating is lower in alexithymics [26]. Yet, the greater activity in the insula indicates a stronger response in the representation of the internal physiologic state in alexithymics [26].

There are overlapping characteristics of deficits in emotional experience and expression between posttraumatic stress disorder (PTSD) and alexithymia [7,8]. Frewen et al. investigated functional neural responses to trauma script imagery associated with severity of alexithymia in a subsample of 26 individuals with PTSD [71]. The TAS-20 score was correlated with increased activity in the right posterior insula and ventral posterior cingulate and decreased activity in the bilateral ventral ACC, ventromedial PFC (vmPFC), anterior insula, and right inferior frontal cortex [71]. Correlation between alexithymic score and right insular activation might be associated with centrally represented body-state mapping of sympathetic arousal, coupled with reduced executive–regulatory cognitive–affective control via the ventral ACC, medial (mPFC), and right inferior frontal cortex [43,45,65,66].

Both Moriguchi’s [22] and Frewen’s [70] studies mentioned above provide further evidence that alexithymia is associated with greater activation in the insular cortex representing interoceptive arousal in response to the internal body-state condition. Interestingly, a recent study measured empathic brain responses in subjects with autism spectrum disorder and control subjects, in which participants anticipated their partner’s pain, which was indicated by an anticipatory cue (colored arrow) [72]. Activity in the anterior insula during the task was negatively correlated with individual differences in TAS score in both subjects with autism spectrum disorder and control subjects [72]. It is quite an interesting phenomenon that real pain and pictures of painful stimuli or a traumatic event induce hyperactivity in the posterior insula, whereas imagination of a partner’s pain provokes less activation in the anterior insula associated with alexithymia. This gradation of activity in the insular cortex accompanying the gradation of the real level of pain appears to explain alexithymic features remarkably. With regard to pain experience, alexithymics may experience pain more strongly at a primitive perceptual level and may not be able to experience pain at a more abstract, conceptual, formal, operational stage. This phenomenon matches the theory of deficient affect development in alexithymia [7,11].

#### Interoceptive awareness

Another notable finding with respect to interoception in alexithymia is interoceptive awareness. Interoceptive awareness tasks measure the ability to perceive (count) one’s heartbeat and represents general sensitivity to visceral processes [73]. Neuroimaging studies have revealed that the anterior insular cortex is of relevance for interoceptive awareness and the rerepresentation and integration of interoceptive bodily signals with higher-order and emotional processes [66]. Interestingly, the heartbeat perception score was inversely correlated with alexithymia in 155 university student subjects, suggesting that alexithymics have less interoceptive awareness [73]. This finding appears to be inconsistent with our results, in which alexithymics show hyperinteroceptive arousal during visceral pain. The discrepancy may be owing to the nature of the tasks. The arousal level of interoception is different; the heartbeat detection task appears to be less arousing than the visceral pain condition. The heartbeat detection task measures attention processing of interoception more and visceral pain processing measures the perception process more. Alexithymics may feel interoceptive arousal more when the bodily state is strongly aroused, or there might be some uncoupling between the attention system and the perception system to bodily signals. Whether activity in the anterior or posterior insular cortex in alexithymia is less or more during the heartbeat detection task should be examined in the future.

#### Chronic pain and alexithymia

Although our results provide evidence that alexithymics may be hypersensitive to interoceptive arousal, recent reports suggest that alexithymia may be related mainly to the affective—rather than the sensory—dimension of chronic pain [20,55]. However, it has been reported that alexithymia is associated with responses to sustained, high-intensity stimuli (pain tolerance level) but not to low-intensity stimuli (pain threshold level) in fibromyalgia [74]. Enhanced sensitivity to unpleasant sensations in alexithymic individuals may become more pronounced during high-intensity stimulation or after prolonged pain stimulation [56]. Association between alexithymia and chronic pain syndromes cannot be explained simply by hypersensitivity to sensory components of painful conditions. Neuroimaging data indicate not only hyperactivity in visceral perception areas, such as the insular cortex, but also hypoactivity in pain-processing, regulatory areas such as prefrontal cortex. Lack of an emotional regulation system might cause hypersensitivity to aversive bodily sensations and prolonged, pain-related affective reactions such as distress. This is a possible mechanism underlying the association between alexithymia and chronic pain.

### Decision-making process based on emotion-guided evaluation in alexithymia

The regulation of emotions has been conceptualized as an integrative, interactional process among the three domains of the emotional response system and with the environment. Affect regulation is a process involving reciprocal interactions between the neurophysiologic, motor-expressive, and cognitive-experiential domains of the emotional response system. Because activation in any one response domain alters or modulates activation in the other two domains, all three domains are involved in the regulation of emotion. Clearly, social bonds, language, dreams, fantasy, play, crying, smiling, and defense mechanisms all play roles in emotional regulation, as does afferent feedback from peripheral autonomic activity and the musculoskeletal system [7]. Affects have important organizing, motivating, and adaptive functions.

Studies that we discuss in this article focus on one domain of the emotional response system, such as response to facial expression for the cognitive-experiential domain or response to visceral pain for the neurophysiologic domain. The true problem in alexithymia, affect dysregulation, is not a simple deficit in one domain of the emotional response system. It is a dysfunction in the interactions among each domain and a dysfunction in the interaction between the emotional response system and the environment. In a third neuroimaging study on alexithymia, we investigated the ability to use emotional signals to guide behavior in social situations.

The Iowa gambling task (IGT) was developed to assess how the decision-making process is influenced by emotionally biased signals, from the observation of patients with damage to the vmPFC [75]. Such decision-making as seen in the IGT presupposes the theory of “somatic markers,” as developed by Damasio [76]. This theory argues that optimal decision-making is not simply the result of rationally, cognitively calculated categorization of gains and losses but is also based on good or bad affective reactions and emotionally guided evaluation. The decision-making deficits found after vmPFC damage owe to an inability to use emotion-based biasing signals generated from the body (somatic markers) when appraising different response options. The reasoning is influenced by crude biasing signals arising from the neural machinery that underlies emotion.

Participants with alexithymia fail to learn an advantageous decision-making strategy, with performance differing significantly from the nonalexithymic group in the last IGT trial [76]. Comparing performance on the IGT and the control task, both groups show brain activation in areas associated with IGT including the PFC, supplementary motor area, inferior parietal lobe, fusiform gyrus, and cerebellum [77]. In addition, nonalexithymics show activation in the bilateral insula and ACC, which have been proposed to house some of the neural substrates of somatic markers. However, alexithymics demonstrate no activation in the insula or ACC but do show activation in the caudate. Comparison between alexithymics and nonalexithymics reveals that alexithymics show lower rCBF in the vmPFC (Brodmann area [BA] 10) and higher rCBF in the caudate and occipital areas during a learning phase of IGT trials [24].

In the context of IGT-related processing, BA10 activity may be associated with the use of internal signals accompanying affective evaluation of stimuli, which is crucial for successful decision-making. The difference in BA10 activity indicates that subjects with alexithymia cannot use brain functions related to emotion-based biasing signals. However, the function of the caudate is to regulate or control impulsivity and disinhibition, and its activity during decision-making has been suggested to be a marker of risk attitude. Individuals with alexithymia may work on the IGT impulsively rather than by using emotion-based signals. This IGT study suggests that individuals with alexithymia may be unable to use feelings to guide their behavior appropriately, in addition to being unable to recognize emotions, which supports previous findings.

There is some behavioral data to support our findings. Undergraduate students (N = 326) performed the standard IGT and a version in which cumulative financial feedback was obscured [78]. Standard learning on the IGT was observed for those scoring low for alexithymia. Higher alexithymia scores were associated with an attenuated learning rate on the IGT, especially under conditions of reduced cognitive information. Those scoring high for alexithymia showed a pattern of response choices that indicated increased risk-taking toward the end of the task [78]. The cognitive skills required to effectively monitor and self-regulate emotions are encompassed in a construct known as emotional intelligence. The emotional intelligence score is inversely correlated with the TAS-20 score [78]. Patients with lesions in structures in the somatic marker circuitry show significantly lower emotional intelligence (high TAS-20 score) and poorer judgment in decision-making, as well as disturbances in social functioning, compared to those with lesions in structures outside the somatic marker circuitry [79].

Taken together, alexithymic individuals may be unable to use emotion for flexible cognitive regulation. Thus, there may be uncoupling or dysfunction in the interaction of the aspects of the emotional response system in alexithymia.

### Cognitive emotional development model and alexithymia

**Figure 1:**
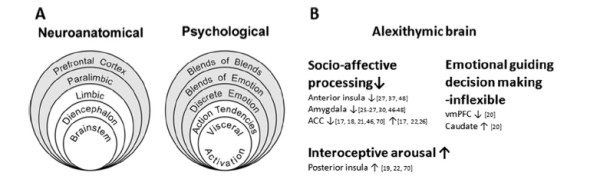
**A: Schema for the five levels of emotional awareness model of Lane and Schwartz**[80]**, Parallels in the hierarchical organization of emotional experience and its neural substrates.** The shell structure is intended to convey that each succeeding level adds to and modulates lower levels but does not replace them. Although each model contains five levels, a one-to-one correspondence between each level in the psychological and neuroanatomical models is not intended. Lower levels with white background correspond to implicit processes. Higher levels with gray background correspond to explicit processes. **B:** Neural machinery characterized by neuroimaging studies on alexithymia. Alexithymics showed weak responses in structures of highly cognitive stages, such as the capacity to feel other’s emotional reactions (socio-affective processing), and stronger responses in primitive stages such as hypersensitivity to visceral pain (interoceptive aroursal). Consequently alexithymics experience inflexible cognitive regulation, owing to impairment of the emotional guiding system. ACC = Anterior cingulate cortex; vmPFC = ventromedial prefrontal cortex.

Lane and Schwartz [80] conceptualized a cognitive-development model for understanding the organization of emotional experience (Figure 1A). In this model, the experience of emotion is hypothesized to undergo structural transformation in a hierarchical developmental sequence of progressive differentiation and integration. There are five levels of emotional organization and awareness in the model: awareness of physical sensations (level 1), action tendencies (level 2), single emotions (level 3), blends of emotions (level 4), and blends of blends of emotional experience (level 5, the capacity to appreciate complexity in the experiences of self and others). The levels are hierarchically related in that functioning at each level adds to and modifies function of the previous levels but does not eliminate them.

Each level of organization corresponds to an identifiable neurobiologic state. Levels 1 and 2 involve implicit processes that are automatic, modular, and cognitively impenetrable. Subcortical structures may participate in the automatic generation of emotional responses associated with absent or diffusely undifferentiated awareness. Neural substrates of level 1 may include the thalamus and hypothalamus (diencephalon) and brainstem. At level 2, the sensorimotor enactive level, the thalamus, amygdala, and ventral striatum would be involved in association with crude distinctions between globally positive and globally negative states. Emotions at level 2 are represented in actions, and the basal ganglia participate in the automatic behavioral displays of emotional expression. Orbitofrontal cortex activity appears to be associated with the perception of bodily sensations that bias behavior. For example, orbitofrontal cortex activity affects behavior by overriding automatic processes in the amygdala.

Levels 3 through 5 involve explicit processes that are influenced by higher cognitive processes, including prior knowledge. They are hypothesized to be mediated by the structures at levels 1 and 2 and by paralimbic structures, including the ACC and insula, and the mPFC. The rostral ACC/mPFC appears to be necessary for the representation of emotion used in conscious cognition. The process of representational redescription is mediated, in part, by structures involved in explicit processing at levels 3 through 5. Because these structures are not uniquely devoted to the processing of emotion, their emotion-related functions are cognitively penetrable. The level of emotional awareness of a given individual may be a function of the degree to which these structures are or are not devoted to processing of emotional information from the internal and external worlds. The hierarchical nature of this anatomic model is parallel to the hierarchical structure of the psychologic model.

## Conclusions

We have conducted three brain imaging studies on alexithymia. The first study displayed weakness of cognitive emotional processing, with less activation in the dACC and anterior insula during the viewing of angry facial expressions. This may represent less function in cognitive mature stages such as levels 3 through 5. The second study showed stronger awareness of visceral sensation in alexithymics, with greater activity in the brainstem, posterior insula, and rostral ACC, accompanying stronger autonomic responses. This indicates stronger responses at primitive stages such as levels 1 and 2. The third study demonstrated greater activation in the caudate (basal ganglia) and less activation in the mPFC in alexithymics during the IGT, which may represent impairment of the interactive, integrative process among the five levels. Thus alexithymics show weak responses in structures necessary for the representation of emotion used in conscious cognition and stronger responses at levels focused on action. Consequently, alexithymics experience inflexible cognitive regulation, owing to impairment of the emotion-guiding system.

The problem in alexithymia has been posited to be deficient development of the cognitive mature stage, which allows some rudimentary form of emotional experience, such as high arousal of bodily sensations. Our neuroimaging studies provide neural evidence for this hypothesis (Figure 1B). A mature emotional system might have a role in alleviating physical sensations and in guiding flexible behavior. Deficient emotional development in alexithymia might lead to hypersensitivity to bodily sensations and unhealthy behaviors, which may be a possible link between alexithymia and psychosomatic disorders.

Alexithymia has been linked with psychiatric conditions, including high-functioning autism/Asperger syndrome and depression, in addition to somatoform disorders [81]. Asperger’s syndrome is characterised by core disturbances in speech and language and problems with affective interaction and has been indicated to have an overlapping character with alexithymia [82,83]. In terms of socio-affective processing, Asperger’s syndrome has similar features to alexithymia. Patients with Asperger’s syndrome showed failure to use information from faces, such as facial affect, eye gaze and facial expression [84], and anomalous activity in the amygdala, fusiform gyrus, and superior temporal gyrus during facial expression recognition [85]. As mentioned above, during anticipation of the partner’s pain, the strength of the signal in the anterior insula was predictive of the degree of alexithymia in both autistic and control groups [72]. However, there was no difference between autistic and control groups after accounting for alexithymia, suggesting that the empathy deficits observed in autism may be due to the large comorbidity between alexithymic traits and autism, rather than representing a necessary feature of the social impairments in autism [72]. In contrast to the reactivity in the amygdala in individuals with alexithymia, many studies have demonstrated hyperactivity in the amygdala in response to negative facial expressions [86-89] and in response to other negative emotional stimuli [90,91] in depressed subjects. Recent studies in adolescents with depression [92,93] and at risk for depression [94] have confirmed that amygdala hyper-responsivity is present in the early stages of illness. Alexithymia has been reported to be associated strongly with depression in both general and clinical populations [6]. It has also been reported that TAS scores increased during depression and decreased after remission [95] and closely linked to concurrent depressive symptoms [96]. However, TAS scores did not predict diagnoses of major depression in a 7-year follow-up study [96]. Co-mobility of depressive disorder cannot explain the neurobiological features of alexithymia. On the other hand, recent study has shown less activity in somatoform patients in the emotional brain circuit, including the bilateral parahippocampal gyrus, the left amygdala, the left superior temporal gyrus, and the insula, during facial emotion recognition. Somatoform patients exhibited increased alexithymia scores (TAS-20) and elevated depression scores (BDI) [97]. Therefore, their neurobiological features to emotional stimuli were more similar to alexithymia.

This article considers the role of emotion in the development of physical symptoms and discusses a possible pathway that we have identified in our neuroimaging studies linking alexithymia with psychosomatic disorders. The Alexithymia construct should be further investigated, not only as psychological problems in affect regulation but also from the viewpoint of an association with physiological symptoms.

## Competing interests

The authors declare that they have no competing interests.

## Authors’ contributions

MK: Study concept and design; interpretation of data; drafting of the manuscript; critical revision of the manuscript for important intellectual content. SF: critical revision of the manuscript for important intellectual content. Both authors read and approved the final manuscript.
